# Second Chern crystals with inherently non-trivial topology

**DOI:** 10.1093/nsr/nwac289

**Published:** 2022-12-20

**Authors:** Xiao-Dong Chen, Fu-Long Shi, Jian-Wei Liu, Ke Shen, Xin-Tao He, C T Chan, Wen-Jie Chen, Jian-Wen Dong

**Affiliations:** School of Physics and State Key Laboratory of Optoelectronic Materials and Technologies, Sun Yat-sen University, Guangzhou 510275, China; School of Physics and State Key Laboratory of Optoelectronic Materials and Technologies, Sun Yat-sen University, Guangzhou 510275, China; School of Physics and State Key Laboratory of Optoelectronic Materials and Technologies, Sun Yat-sen University, Guangzhou 510275, China; School of Physics and State Key Laboratory of Optoelectronic Materials and Technologies, Sun Yat-sen University, Guangzhou 510275, China; School of Physics and State Key Laboratory of Optoelectronic Materials and Technologies, Sun Yat-sen University, Guangzhou 510275, China; Department of Physics, The Hong Kong University of Science and Technology, Hong Kong, China; School of Physics and State Key Laboratory of Optoelectronic Materials and Technologies, Sun Yat-sen University, Guangzhou 510275, China; School of Physics and State Key Laboratory of Optoelectronic Materials and Technologies, Sun Yat-sen University, Guangzhou 510275, China

**Keywords:** topological photonics, synthetic dimension, photonic crystals, dislocation mode

## Abstract

Chern insulators have been generalized to many classical wave systems and thereby lead to many potential applications such as robust waveguides, quantum computation and high-performance lasers. However, the band structure of a material can be either topologically trivial or non-trivial, depending on how the crystal structure is designed. Here, we propose a second Chern crystal in a four-dimensional parameter space by introducing two extra synthetic translation dimensions. Since the topology of the bulk bands in the synthetic translation space is intrinsically non-trivial, our proposed four-dimensional crystal is guaranteed to be topologically non-trivial regardless of the crystal's detailed configuration. We derive the topologically protected modes on the lower dimensional boundaries of such a crystal via dimension reduction. Remarkably, we observe the one-dimensional gapless dislocation modes and confirm their robustness in experiments. Our findings provide novel perspectives on topologically non-trivial crystals and may inspire designs of classical wave devices.

## Teaser

Featuring with the gapless dislocation and corner modes, second Chern crystals with inherently nontrivial topology were demonstrated in the four-dimensional space with the introduction of synthetic translation dimension.

## INTRODUCTION

Topological phases of matter and their extensions to classical wave systems have revolutionized our understanding of the edge states of crystals [1–14]. Boundary properties are closely related to the bulk band topology, which arises from the connection between Bloch wavefunctions characterized by a global topological invariant. One prominent example is the Chern insulator, which can support the chiral edge states and exhibit quantized Hall conductance [1,2]. Its associated non-trivial band topology is characterized by the first Chern number, which counts the phase winding of eigen wavefunctions along the boundary of the two-dimensional (2D) Brillouin zone in the momentum space [15,16]. In principle, the Chern numbers depend on the structural parameters of bulk crystals and can be any integer (trivial or non-trivial), and non-zero values of Chern number can be obtained if the crystal structures are carefully designed.

However, discussion of the band topology is not only restricted to the Bloch momentum space but can also be expanded into the synthetic space where the wavefunctions depend on system parameters such as the external electric or magnetic field, the geometric size and so on [17–25]. Clearly, the synthetic space enables us to study high-dimensional topological states without the limitations of real-space dimensionality. A prototypical example is the four-dimensional (4D) Chern insulator [26,27], i.e. the 4D generalization of 2D quantum Hall effect, based on the construction of a 4D lattice system in synthetic space. The 4D Chern insulator is characterized by the second Chern number defined in a 4D (pseudo-) momentum space. Since classical wave systems can be achieved by high-quality samples with tailorable band dispersions characterized effortlessly using well-established measurement techniques [28–31], they are nice platforms for investigating high-dimensional topological physics in synthetic space and the associated potential applications [32–43]. Indeed, 4D Chern insulators have already been realized in various types of systems, such as 2D acoustic crystals [39], 2D optical lattices [40] and 3D electric circuits [42,43]. However, it is still quite challenging to design these complex periodic lattices or structures.

Here, we report the realization of a second Chern crystal in the 4D momentum space spanned by a 2D Bloch momentum space and a 2D synthetic translation space. Different from the aforementioned 4D Chern insulators, topology of the proposed 4D crystal is intrinsically non-trivial regardless of the detailed crystal parameters. Each 4D bulk band is characterized by a second Chern number of +1 due to its unique connection between wavefunctions. This topology leads to the gapless 2D surface modes emerging as a result of dimension reduction. Additionally, in the experiment we observe the existence and robustness of gapless 1D vortex line modes manifested by 0D dislocation modes in real space. These low-dimensional boundary modes originate from the intrinsically non-trivial topology of the synthetic translation space, rather than the specific parameters of the crystals. Therefore, the boundary modes universally persist for any crystals with arbitrary lattices, unit cell geometries or material parameters. The observed phenomenon ensured by topological band theory will reform our design philosophy with regard to photonic crystals, and pave the way for inventing novel classical wave devices based on the synthetic translation space.

## RESULTS

**Figure 1.**
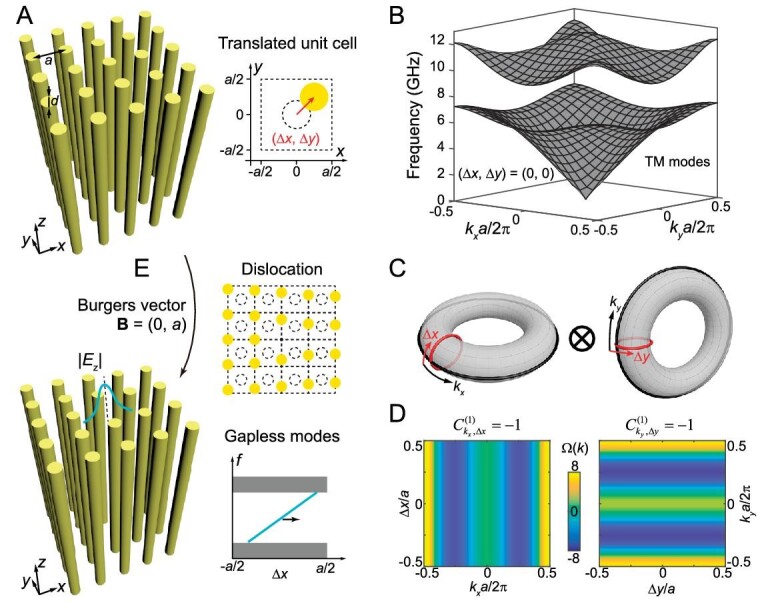
Second Chern crystal in 4D synthetic translation space, and its gapless chiral dislocation modes. (A) Left: schematic of a perfect square lattice of dielectric rods (yellow) in air. Right: a translated unit cell where the rod is translated by (Δ*x*, Δ*y*) from the origin. Parameters: lattice constant *a* = 14 mm, diameter and relative permittivity of rod *d* = 5.6 mm and ϵ = 8.0. (B) Bulk bands of transverse magnetic (TM) modes of the 2D PC with (Δ*x*, Δ*y*) = (0, 0) in the (*k_x_, k_y_*) space, along with a complete band gap spanning 7.24 to 9.63 GHz. (C) Schematic of the 4D Brillouin zone spanned by (*k_x_*, Δ*x, k_y_*, Δ*y*). (D) Berry curvatures of the first lowest bulk band in the (*k_x_*, Δ*x*) space with *k_y_* = 0 and Δ*y* = 0 (left) and in the (*k_y_*, Δ*y*) space with *k_x_* = 0.8π/*a* and Δ*x* = −0.3*a* (right), giving non-zero first Chern numbers in the corresponding 2D subspaces. (E) Schematic of the dislocation lattice characterized by the Burgers vector of **B** = (0, *a*). A gapless dislocation mode, which is localized around the origin, is expected to traverse the bulk gap.

Instead of plunging into the general proof of our argument, we start with a specific example of square lattice photonic crystal (PC). As depicted in Fig. 1A, a square array of dielectric rods (relative permittivity of ϵ = 8.0) are considered. Figure 1B shows the transverse magnetic (TM) bulk bands in the 2D momentum space. A complete band gap exists between the first and second bulk bands from 7.24 to 9.63 GHz. To check the topological feature of this band gap, we calculate the first Chern number of the first bulk band by integrating Berry curvature *B*(*k_x_, k_y_*) in the 2D Brillouin zone. As both time reversal symmetry and inversion symmetry are preserved, Berry curvature *B*(*k_x_, k_y_*) vanishes everywhere in the (*k_x_, k_y_*) momentum space. Therefore, the first bulk band is characterized by $C_{{k}_x,{k}_y}^{(1)} = 0$ and the 2D complete band gap from 7.24 to 9.63 GHz is topologically trivial.

In order to discuss a 4D band structure, we need to define a 4D momentum space by introducing synthetic dimensions. Here we assume that the dielectric rod is translated away from the unit cell center by (Δ*x*, Δ*y*) (right panel of Fig. 1A). The two translation parameters can be deemed as external parameters of the system, just like *k_x_* and *k_y_*. Since both Δ*x* and Δ*y* fall in a finite and closed parameter space, i.e. −0.5*a* < Δ*x* ≤ 0.5*a* and −0.5*a* < Δ*y* ≤ 0.5*a*, they serve as two additional Bloch momenta. In addition, they are independent from the Bloch momenta *k_x_* and *k_y_*. Then we can define a 4D parameter space (*k_x_*, Δ*x, k_y_*, Δ*y*) and investigate its 4D band structure with eigenfrequency of *ω_n_*(*k_x_*, Δ*x, k_y_*, Δ*y*). It is obvious that the eigenfrequencies of bulk modes should not depend on Δ*x* or Δ*y*. Therefore the frequency regime from 7.24 to 9.63 GHz is a 4D complete band gap in the band structure of *ω_n_*(*k_x_*, Δ*x, k_y_*, Δ*y*). To study the topological feature of this 4D band gap, we need to calculate the second Chern number of the lowest bulk band, which is given by:


(1) \begin{eqnarray*} {C}^{(2)} &=& \frac{1}{{32{\pi }^2}}\int{{\varepsilon }_{lmno}{B}_{lm}}({k}_x,\Delta x,{k}_y,\Delta y)\times{B}_{no}\\ &&{({k}_x,\Delta x,{k}_y,\Delta y)d{k}_xd\Delta xd{k}_yd\Delta y},\\ \end{eqnarray*}

where ${\varepsilon }_{lmno}$ is the Levi-Civita symbol, and Berry curvature ${B}_{lm}({k}_x,\Delta x,{k}_y,\Delta y) = {\partial }_l{A}_m({k}_x,\Delta x, {k}_y,\Delta y) - {\partial }_m{A}_l({k}_x,\Delta x,{k}_y,\Delta y)$ is written in terms of the Berry connection ${A}_l({k}_x,\Delta x,{k}_y, \Delta y) = \langle {u({k}_x,\Delta x,{k}_y,\Delta y)} |{\partial }_l| {u({k}_x,\Delta x,{k}_y,\Delta y)} \rangle $. Here, $u({k}_x,\Delta x,{k}_y,\Delta y)$ is the periodic wavefunction and each index *l, m, n, o* takes values of *k_x_*, Δ*x, k_y_* or Δ*y*. Taking into account the symmetries of the Berry curvature, i.e. ${B}_{lm} = - {B}_{ml}$, the second Chern number can be rewritten as ${C}^{(2)} = C_{{k}_x,\Delta x}^{(1)}C_{{k}_y,\Delta y}^{(1)} - C_{{k}_x,\Delta y}^{(1)}C_{{k}_y,\Delta x}^{(1)} - C_{{k}_x,{k}_y}^{(1)}C_{\Delta x,\Delta y}^{(1)}$ [44]. According to this simplified expression, the second Chern number can be obtained by multiplying the first Chern numbers in each 2D subspace. Figure 1D plots the numerical Berry curvatures in the (*k_x_*, Δ*x*) and (*k_y_*, Δ*y*) subspaces, whose integrals over the 2D Brillouin zone ($C_{{k}_x,\Delta x}^{(1)}$ and $C_{{k}_y,\Delta y}^{(1)}$) are both −1. The Δ*x*-independent (Δ*y*-independent) Berry curvatures on the left (right) panel are distinctly different from those of 2D magnetic PCs [45,46] (more discussion in Supplement A). Although these two first Chern numbers are numerically demonstrated to be −1 in a square PC with specific parameters (rod radius, permittivity, etc.), this conclusion universally applies and will be proven below. We take $C_{{k}_x,\Delta x}^{(1)}$ as an example and consider the 2D (*k_x_*, Δ*x*) cut plane in 4D k space with constant *k_y_* and Δ*y*. Since the 2D crystal with translation parameters (Δ*x*, Δ*y*) is related to the 2D crystal with (0, Δ*y*) by a translation of Δ*x*, the eigen wavefunctions of these two crystals are also related. Suppose the wavefunction at 4D momenta (*k_x_*, 0, *k_y_*, Δ*y*) is ${u}_{{k}_x,0,{k}_y,\Delta y}(x,y)$, then the wavefunction at (*k_x_*, Δ*x, k_y_*, Δ*y*) should be ${u}_{{k}_x,\Delta x,{k}_y,\Delta y}(x,y) = {u}_{{k}_x,0,{k}_y,\Delta y}(x - \Delta x,y)$. Note that this relation universally applies and does not depend on the specific parameters of the crystal (rod radius, permittivity, etc.). By employing this relation between wavefunctions under different translations, one can prove that $C_{{k}_x,\Delta x}^{(1)} \equiv - 1$ (Supplement B). The Berry curvatures in the other four 2D subspaces are also numerically calculated, and their integrals are all 0 (Fig. S2). It can be proven that $C_{{k}_x,\Delta y}^{(1)}$, $C_{{k}_y,\Delta x}^{(1)}$ and $C_{\Delta x,\Delta y}^{(1)}$ are inherently zero, i.e. $C_{{k}_x,\Delta y}^{(1)} = C_{{k}_y,\Delta x}^{(1)} = C_{\Delta x,\Delta y}^{(1)} \equiv 0$, which does not depend on the specific form of wavefunctions (see details in Equations (B6)–(B8) in Supplement B). In addition, $C_{{k}_x,{k}_y}^{(1)}$ vanishes under time reversal symmetry, i.e. $C_{{k}_x,{k}_y}^{(1)} = 0$. Therefore, the second Chern number of the first bulk band should be:


(2) \begin{eqnarray*} {C}^{(2)} &\equiv &( - 1) \times ( - 1) - (0) \times (0) - (0) \times (0)\\ & \equiv & 1. \end{eqnarray*}

As the second Chern number of one band gap is equal to the sum of the second Chern number of bulk bands below the gap, the first band gap between the first and second bulk bands is characterized by a gap second Chern number of +1, indicating a non-trivial second Chern crystal. In addition, as the theoretical derivation of the second Chern number is independent of specific wavefunctions, each single bulk band in the 4D (*k_x_*, Δ*x, k_y_*, Δ*y*) space is also characterized by a second Chern number of +1. In this sense, any 2D crystal with a complete band gap is a topologically non-trivial second Chern crystal, and the gap second Chern number of each band gap is equal to the number of bulk bands below the gap. Note that 1D crystals equipped with one synthetic translation dimension have been proposed and investigated, and they exhibit topological properties equivalent to a 2D quantum Hall system [37,38]. However, the (D-2) dimensional boundary modes and the associated higher-order topological physics have not been investigated. And the higher-order topology with (D-3) dimensional modes cannot be achieved in the 2D synthetic system.

**Figure 2.**
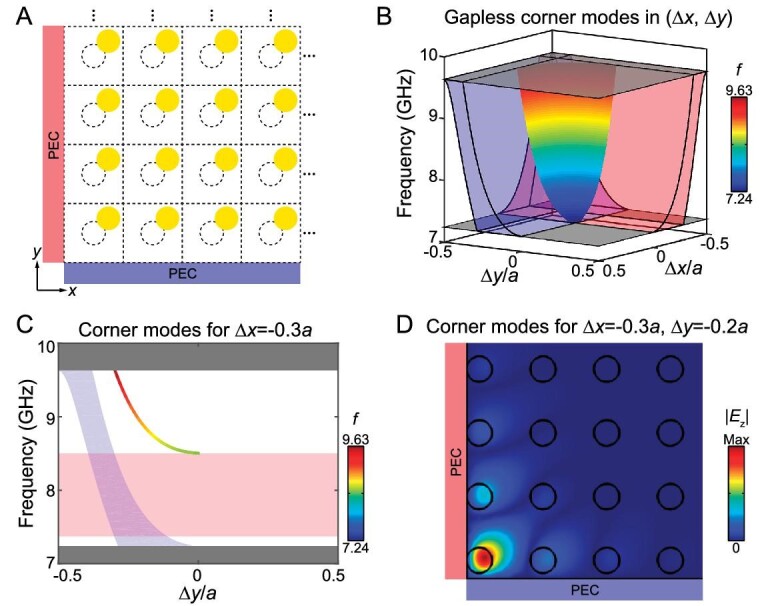
2D topological surface modes manifested as 0D corner modes in real space. (A) Schematic of the corner between the 2D PC with (Δ*x*, Δ*y*) and two perfect electric conductors (PECs) on the left and the bottom. (B) 2D band dispersion of gapless corner modes (shaded in colors) as a function of (Δ*x*, Δ*y*). The projected bands of 3D hypersurface modes for two PEC boundaries are shaded in red and blue. Two transparent gray squares mark the lower and upper frequency edges of the band gap. (C) Corner mode dispersion on the cut plane at Δ*x* = −0.3*a* in (B). (D) Field pattern |*E_z_*| of the corner mode for the crystal with (Δ*x*, Δ*y*) = (−0.3*a*, −0.2*a*).

**Figure 3.**
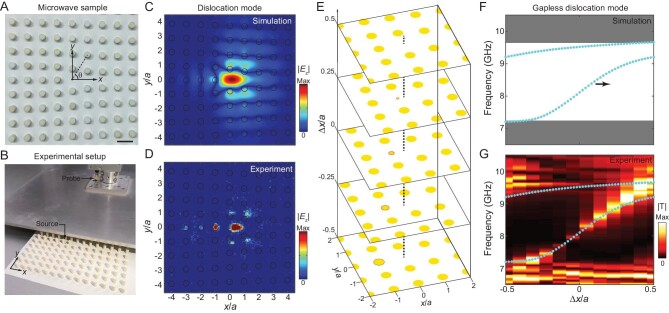
1D vortex line modes manifested as 0D dislocation modes in real space. (A) Microwave sample of the dislocation lattice (only the zoomed-in central part is shown). The translations of rods obey (0.5*a, θa*/2*π*), leading to a dislocation lattice with a Burgers vector of **B** = (0, *a*). Scale bar: 14 mm. (B) Set-up for the near-field scanning measurement. The top metallic plate is shifted to show the inside rod array. (C and D) Simulated and measured field patterns |*E_z_*| of the dislocation mode. (E) 3D schematic of a series of dislocation lattices with different Δ*x*. The dashed line locates the vortex point. Five representative dislocation lattices (with Δ*x* = −0.5*a*, −0.25*a*, 0, 0.25*a*, 0.5*a*) are plotted as examples. To preserve the continuity of lattices with Δ*x* = 0.5*a* and Δ*x* = −0.5*a*, the diameter of the first rod to the left of the origin (outlined in red) is set as *d*_0_ = −(Δ*x*/*a*–0.5)*d*. (F and G) Simulated and measured eigenfrequency spectra in which gapless dislocation bands are observed. Simulated band dispersion (cyan) is added in (G) for the comparison.

**Figure 4.**
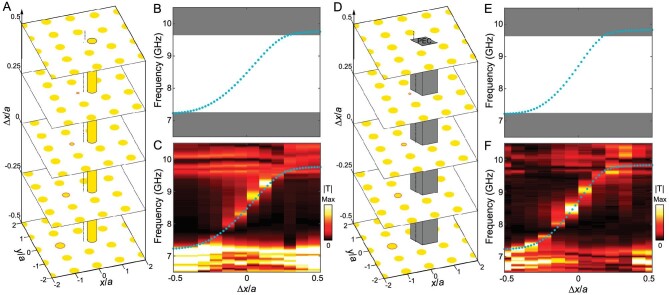
Robustness of gapless dislocation modes. (A and D) 3D schematic of the 1D vortex line with (A) an additional dielectric rod and (D) a PEC defect on the right of the origin. (B and C, and E and F) Simulated and measured frequency spectra for (B and C) the samples with an additional dielectric rod and (E and F) the samples with a PEC defect. In both cases, although the band dispersions are altered, the gapless chiral feature is preserved.

The inherently non-trivial topology of this second Chern crystal would manifest itself as the gapless boundary modes when the 4D bulk crystal is truncated to construct lower dimensional boundaries. As a typical example, 1D gapless dislocation modes can be obtained by truncating the 4D crystal in three dimensions (Fig. 1E), which is clarified in detail in Figs 3 and 4. Topological boundary modes also exist when the 4D crystal is truncated in one dimension to form a 3D hypersurface (leading to 1D edge modes in real space, which are presented in Supplement C), or when it is truncated in two dimensions to form a 2D surface (leading to 0D localized corner modes in real space). Here we truncate the crystal in both the *x* and *y* directions and form a corner bounded by two perfect electric conductors (PECs), as illustrated in Fig. 2A. Figure 2B shows the dispersion of 2D surface modes (shaded in colors) in the (Δ*x*, Δ*y*) space. The 3D hypersurface modes for the left and the bottom boundaries are shaded in red and blue, respectively. Outside the projection of two hypersurface bands are a set of gapless surface modes localized at the corner, which are guaranteed by the non-zero first Chern numbers of hypersurface modes for the left and bottom boundaries (i.e. $C_{{k}_y,\Delta y}^{(1)} = - 1$ and $C_{{k}_x,\Delta x}^{(1)} = - 1$). Here, we take the cut plane at Δ*x* = −0.3*a* as an example (Fig. 2C) and consider the hypersurface modes located at the left boundary (Fig. S3(a)). Since the hypersurface band in (*k_y_*, Δ*y*) space is well separated from bulk bands, its first Chern number is well defined and equal to −1 (see Supplement C). Accordingly, a gapless corner mode dispersion (colored curve in Fig. 2C) exists above the projected hypersurface band (translucent red in Fig. 2C). This argument also applies for any cut plane with fixed Δ*y* and therefore the dispersion of corner modes is an arched sheet in the (Δ*x*, Δ*y*) space (colored in Fig. 2B) connecting two projected hypersurface bands (red and blue in Fig. 2B). The |*E_z_*| field of one representative corner mode at (Δ*x*, Δ*y*) = (−0.3*a*, −0.2*a*) is plotted in Fig. 2D, showing strong field confinement around the corner. In this work, gapless hypersurface modes and gapless surface modes are found between the second Chern crystal and the PEC. Note that gapless boundary modes also exist at the interface between the second Chern crystal and other trivial insulators (see more in Supplement D), and here the PEC is used as a representative example of trivial insulators.

Another manifestation of the non-trivial topology of a second Chern crystal is the gapless dislocation modes along a 1D vortex line [47,48]. To see this, we first fabricate a microwave sample where the translation of each rod away from its lattice point is (Δ*x*, Δ*y*) = (0.5*a, θa*/2*π*) (Fig. 3A). Here, Δ*x* is a constant while Δ*y* varies as the azimuthal angle *θ*, leading to a dislocation near the origin with a Burgers vector of ${{\bf B}} = (0,a)$ (upper-right panel of Fig. 1E). The dislocation point is assumed to lie at the origin, as depicted by the black solid point. The numerical simulation in Fig. 3C shows that such a dislocation supports an in-gap mode localized around the origin, at the frequency of *f* = 9.2 GHz. To demonstrate this, we carry out a near-field scanning measurement (Fig. 3B). A source antenna is inserted through the metallic substrate to excite the eigenmodes and a probe antenna is mounted on a translational stage to measure the spatial field distributions. The measured |*E_z_*| field exhibits strong localization around the origin (Fig. 3D), in good agreement with the simulated one. By changing Δ*x* and considering it as a pseudo momentum, frequencies of dislocation modes evolve as a gapless band and exhibit a chiral dispersion. Here, ‘chiral’ means that the eigenfrequency of the dislocation mode monotonically increases or decreases as a function of Δ*x*. The slope of dislocation band is just the parameter dependence of eigenfrequency, rather than group velocity. Thus, these dislocation modes do not really ‘propagate’ in real space as those in 3D crystals do. The gapless dislocation mode is equivalent to a 1D vortex line state of a 4D Chern insulator. We take five representative dislocation lattices (with Δ*x* = −0.5*a*, −0.25*a*, 0, 0.25*a*, 0.5*a*) as examples (Fig. 3E). For each lattice with a fixed Δ*x*, a vortex point lies at the origin, around which are rods with different Δ*y*. To preserve the continuity between lattices with Δ*x* = 0.5*a* and Δ*x* = −0.5*a*, the diameter of the first rod on the left of the origin (outlined in red) is set as *d*_0_ = −(Δ*x*/*a*–0.5)*d*. Figure 3F plots the eigenfrequency spectra of in-gap dislocation modes as a function of Δ*x* (highlighted in cyan). It exhibits a gapless dispersion traversing the whole band gap. The eigenfrequency of dislocation mode increases as Δ*x*, and this is consistent with the second Chern number of +1. To demonstrate the chiral gapless dispersion, we carry out a pump-probe transmission measurement [29,49] by placing the source antenna near the third rod on the left of the origin and the probe antenna at (10.5 mm, 0) with respect to the origin. The measured frequency dispersion of the dislocation modes (bright color) agrees well with the calculated one (Fig. 3G). Note that the number and the monotonous Δ*x*-dependence of gapless dislocation modes are in correspondence with the Burgers vector. One can realize more dislocation bands by constructing a dislocation lattice with a longer Burgers vector (see more in Supplement E).

It is noteworthy that the topologically protected cavity modes or corner modes have been investigated recently in various systems [50–52]. These topological mid-gap modes are robust against disorder that does not close the band gap. In other words their eigenfrequencies are unchanged when the artificial symmetries are preserved. But in principle, these modes would inevitably merge into the bulk band as long as the perturbation is large enough or breaks the artificial symmetry. Similarly, this is true for our system if we only focus on one dislocation lattice with a fixed Δ*x*. But if we examine the whole spectrum as a function of Δ*x*, we will find that gapless dislocation mode dispersion always spans the band gap, and the number of gapless bands is unchanged. To see this, we consider the same dislocation lattice as that in Fig. 3A but with an additional dielectric rod (with diameter of 5.6 mm) or a rhombus PEC block (with side length of 9 mm) (Fig. 4A and D). Centers of the additional rod and PEC block are fixed at (5 mm, 0). In both cases, with introduced defects, the eigenfrequency of the dislocation mode of the lattice with Δ*x* = 0.5*a* is shifted from 9.2 to 9.75/9.83 GHz, merging into the frequency ranges of bulk bands. But from the overall view of the spectra in Fig. 4B and E, there is one gapless band with its frequency increasing as Δ*x*. Although the spectra for both defected cases are altered, the gapless feature is preserved by the non-trivial topology of the proposed second Chern crystal. This is also confirmed by our experimental results (Fig. 4C and F). Note that there are always topologically protected dislocation modes in spite of the strength of defects (see more in Supplement F).

## CONCLUSION

We have successfully achieved the second Chern crystal in the 4D synthetic translation space spanned by a 2D Bloch momentum space and a 2D translation parameter space. Its band structure shows non-trivial topology independent of the structural parameters and geometry of the unit cell. The intrinsically non-trivial feature within this particular 4D momentum space guarantees the existence of chiral boundary modes (e.g. 2D surface modes and 1D vortex line modes). In particular, 1D vortex line modes manifested by 0D dislocation modes in real space were experimentally observed, as well as their robustness against defects. Note that in the 4D synthetic momentum space, the non-trivial second Chern number universally applies to bulk bands of a crystal with any lattices, unit cell geometries or material parameters. Two examples, i.e. the oblique lattice of dielectric rods in the air and the triangular lattice of air rods in the dielectric background, are given in Supplement G. In both cases, there exist the gapless corner modes and gapless dislocation modes.

## METHODS

### Simulation

All numerical simulations are performed with COMSOL Multiphysics. An eigenfrequency study is used to calculate band structures. To simulate 3D hypersurface modes in Fig. S3, we consider a supercell with 10 × 1 unit cells in the *x*–*y* plane and apply the same translation (Δ*x*, Δ*y*) to all rods. Floquet periodic boundary conditions with a wave vector of *k_y_* are applied on the upper and lower boundaries, while a PEC boundary condition is applied on the left boundary. To simulate 2D surface modes in Fig. 2, we consider a structure with 10 × 10 unit cells in the *x*–*y* plane and apply the same translation (Δ*x*, Δ*y*) to all rods. PEC boundary conditions are applied on the left and bottom boundaries. To simulate 1D vortex line modes (i.e. 0D dislocation modes in real space) in Fig. 3, we construct a lattice structure with a size of 10*a* × 11*a*. To mimic the dislocation at the origin with a Burgers vector of (0, *a*), the translation of each rod away from its lattice point is (Δ*x*, Δ*y*) = (0.5*a, θa*/2*π*). Here, Δ*x* is a constant while Δ*y* varies as the azimuthal angle *θ*. To test the robustness of gapless dislocation modes in Fig. 4, an additional rod with a diameter of 5.6 mm is added at (5 mm, 0) with respect to the origin. In another case, a PEC block with a rhombus shape and a side length of 9 mm is inserted at (5 mm, 0). All models for these numerical simulations are summarized in Fig. S13.

### Microwave experiment

A 2D square lattice of dielectric rods with the lattice constant *a* = 14 mm is fabricated in the experiment. The dielectric rods are made of ceramic with a relative permittivity of *ϵ* = 8. The height and diameter of rods are *h* = 17 mm and *d* = 5.6 mm. As shown in Fig. 3B, all rods are sandwiched between two parallel metal plates where the zeroth transverse magnetic waveguide modes (electric fields are perpendicular to the *x*–*y* plane) behave like the transverse magnetic modes of 2D PCs. In the experiment, each dislocation sample contains 14 × 13 unit cells whose size is large enough to guarantee the localization of dislocation modes (Fig. S14). For all samples, the diameter of the first rod to the left of the origin is set as *d*_0_ = −(Δ*x*/*a*–0.5)*d*. As shown in Figs S14(b) and (c), the additional rod and PEC defects are introduced and centered at (5 mm, 0) to test the robustness of gapless dislocation modes.

The localization of dislocation modes is observed by carrying out a near-field scanning measurement (Fig. 3B). A source antenna is inserted through the metallic substrate to excite the eigenmodes. A probe antenna is fixed on the top metal plate, which is mounted on a 2D motorized translation stage (LINBOU NFS03) to scan the field pattern. A 1 mm air gap between the dielectric rod array and the top metal plate is reserved to allow the top metal plate to move. The input and detected signals are emitted and collected by the vector network analyzer (Keysight E5071C). We measure the band dispersion of dislocation modes by carrying out a pump-probe transmission measurement. During the measurement, the source antenna is always placed next to the right side of the third rod on the left of the origin (marked by red circles in Fig. S14). The probe antenna is placed at (10.5 mm, 0) (marked by blue circles in Fig. S14). One example of the measured transmission spectrum for the dislocation sample with Δ*x* = −0.04*a* (i.e. the diameter of the first rod on the left of the origin is *d*_0_ = 3 mm) is shown in Fig. S15(a). Within the frequency range of the band gap, the transmission peak indicates the existence of the localized dislocation mode. By summarizing all the transmission spectra, we can obtain the band dispersion of dislocation modes (Fig. S15(b), i.e. Fig. 3G).

## Supplementary Material

nwac289_Supplemental_File
