# Supplementary material for: Second Chern crystals with inherently non-trivial topology
Source: Natl Sci Rev. 2022 Dec 20;10(8):nwac289. doi: 10.1093/nsr/nwac289 (PMC10306366; doi:10.1093/nsr/nwac289)
Supplement: nwac289_Supplemental_File [file nwac289_supplemental_file.pdf]

Supplemental Material for

Second Chern crystals with inherently nontrivial topology

Xiao-Dong Chen[1], Fu-Long Shi[1], Jian-Wei Liu[1], Ke Shen[1], Xin-Tao He[1], C. T. Chan[2],
Wen-Jie Chen[1,*], and Jian-Wen Dong[1,*]

1 School of Physics & State Key Laboratory of Optoelectronic Materials and Technologies, Sun Yat-sen University, Guangzhou 510275, China.

2 Department of Physics, The Hong Kong University of Science and Technology, Clear Water Bay, Kowloon, Hong Kong, China

[*]Corresponding author: chenwenj5@mail.sysu.edu.cn, dongjwen@mail.sysu.edu.cn

Contents:

A. Nonzero Berry curvatures in 2D synthetic translation space and 2D reciprocal space

B. Detailed derivation of second Chern number

C. Gapless 3D hypersurface modes

D. Gapless boundary modes at interface between second Chern crystal and trivial insulators

E. Gapless dislocation modes under different Burgers vectors

F. Existence of gapless dislocation modes in spite of the strength of defect

G. Gapless corner modes and dislocation modes for the crystals with other lattice types

H. Details in the numerical simulation and experimental measurement

A: Nonzero Berry curvatures in 2D synthetic translation space and 2D reciprocal space

In this section, we compare the nonzero Berry curvatures in 2D synthetic translation space and 2D reciprocal space. As illustrated in the main text, the dielectric rod of a 2D square lattice of PC is translated away from the origin by (Δx, Δy) [Fig. S1(a)]. Combining this 2D translation space and the 2D Bloch momentum space, we can define a 4D Brillouin zone. For any constant k_y and Δy, we have a 2D subspace which is spanned by k_x and Δx [Fig. S1(b)]. Figure S1(c) shows the bulk bands along the high symmetry k-lines in the (k_x, Δx) space by keeping $k_y = 0$ and $\Delta y = 0$. Clearly, a complete band gap exists between the first and second bulk bands. The topology of this band gap is determined by the first Chern number of the bulk band below it. Therefore, we calculate the Berry curvature of the first bulk band within the (k_x, Δx) space [Fig. S1(d)]. Berry curvatures in this 2D subspace are Δx-independent, since the wavefunction at (k_x, Δx) can be translated by that at (k_x, 0), i.e. $u_{k_x,\Delta x}(x) = u_{k_x,0}(x-\Delta x)$ which does not depend on the specific form of wavefunction. This Berry curvature distribution is distinctly different from that of 2D magnetic photonic crystals (MPCs). To see this, we present a 2D square lattice of MPC for a detailed comparison. Its unit cell consists of a circular yttrium-iron-garnet rod with the radius of $0.11a$ in air [Fig. S1(e)]. The transverse magnetic modes are considered, and the constitutive parameters of the rod are $\varepsilon_r = 15$ and $\mu_r = \begin{bmatrix} 3.5 & -3i \\ 3i & 3.5 \end{bmatrix}$. To simplify the problem, we assume these artificial parameters and neglect material dispersion which will not affect the band topology. For this MPC, the considered 2D closed space is spanned by k_x and k_y, i.e., the 2D reciprocal space [Fig. S1(f)]. Figure S1(g) shows the bulk band dispersions along with their first Chern numbers. The second bulk band is characterized by the first Chern number of -1 as those bulk bands in Fig. S1(c). However, their Berry curvature distributions are distinctly different [Figure S1(h)]. The Berry curvatures in Fig. S1(h) are localized around the Brillouin zone corner (M point) and they are

k-independent, showing a striking contrast to the Δx-independent Berry curvatures in Fig. S1(d). In addition, the first Chern number of the first band of this MPC is 0. It suggests that the first Chern number in 2D reciprocal space depends on the specific form of wavefunction. On the other hand, if we change the parameters of the 2D PCs in 4D synthetic space, their band dispersions and wavefunctions will change. But their first Chern number in the 2D (k_x, Δx) subspace (or 2D (k_y, Δy) subspace) remain -1 [see derivation in Supplement B].

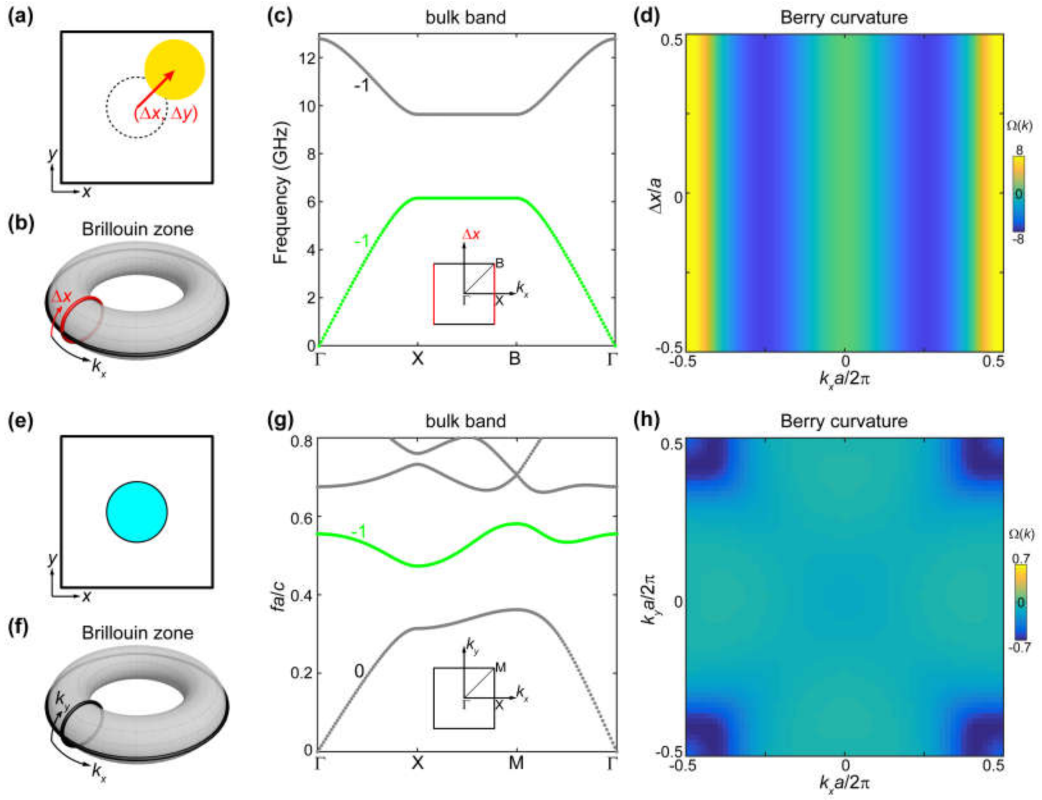

FIG. S1. Nonzero Berry curvatures in 2D synthetic translation space and 2D reciprocal space. (a) The unit cell of 2D translated PC whose central rod is translated by (Δx, Δy) from the origin. (b) Schematic of the 2D Brillouin zone spanned by k_x and Δx. (c) Bulk bands along the high symmetry k-lines of the 2D (k_x, Δx) space for the translated PC with $k_y = 0$ and $\Delta y = 0$. The first bulk band with a nonzero first Chern number of -1 is highlighted in green. (d) Berry curvatures of the first bulk band. (e) The unit cell of a square lattice of MPC which consists of a yttrium-iron-garnet rod (cyan) in the air. (f) Schematic of the 2D Brillouin zone spanned by k_x and k_y. (g) Bulk bands of the MPC whose parameters are given in the text. The second bulk band with a nonzero first Chern number of -1 is highlighted in green. (h) Berry curvatures of the second bulk band.

Lastly, we summarize the Berry curvatures for the 2D translated PC in all six 2D subspaces [Fig. S2]. Clearly, nonzero Berry curvatures are found in the (k_x, Δx) and (k_y, Δy) subspaces, and their integral over the Brillouin zone indicate the nonzero first Chern numbers of $C^{(1)}_{k_x,\Delta x} = C^{(1)}_{k_y,\Delta y} = -1$. On the contrary, zero Berry curvatures are found in other four 2D subspaces, and they lead to the zero first Chern numbers of $C^{(1)}_{k_x,k_y} = C^{(1)}_{k_x,\Delta y} = C^{(1)}_{k_y,\Delta x} = C^{(1)}_{\Delta x,\Delta y} = 0$.

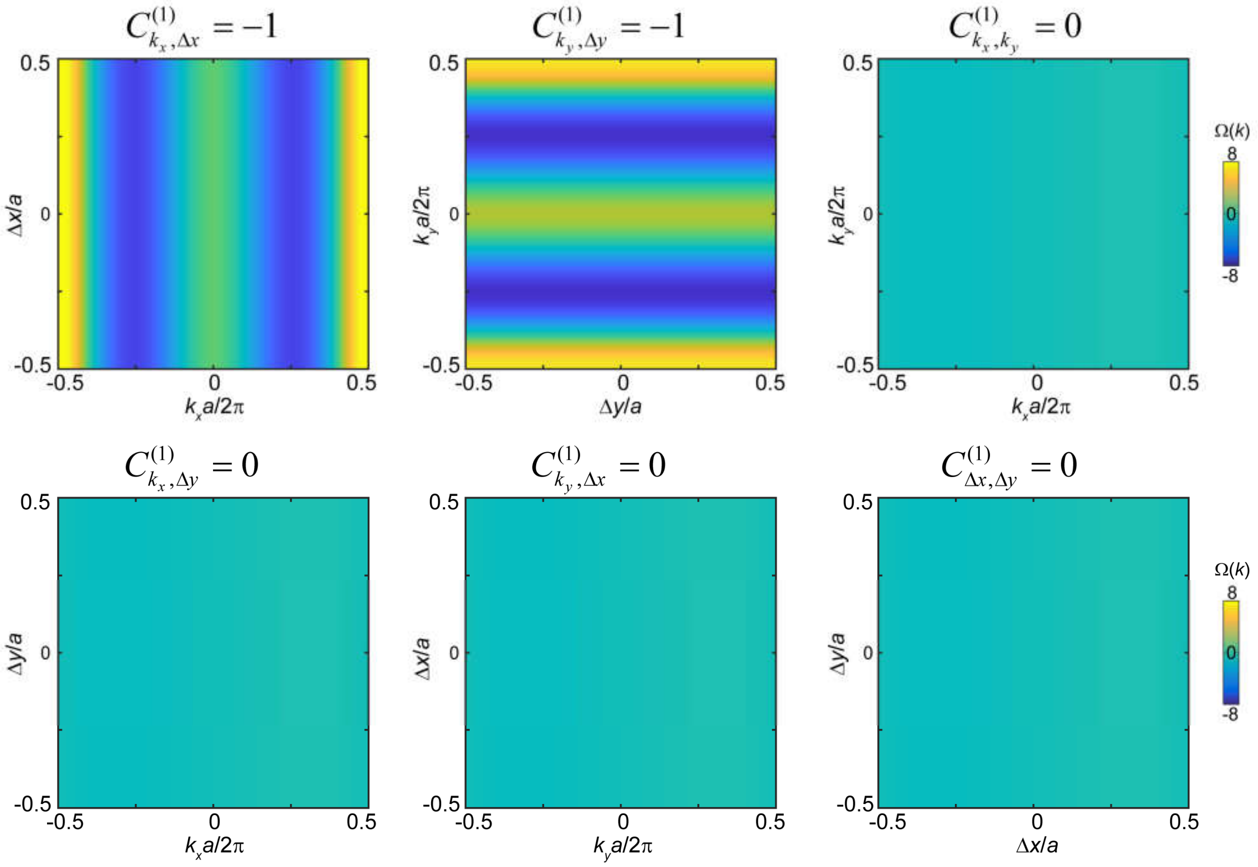

FIG. S2. Berry curvatures in six 2D subspaces. Berry curvatures are nonzero in the (k_x, Δx) and (k_y, Δy) subspaces, while the ones in other four 2D subspaces are zero.

B: Detailed derivation of second Chern number

In this section, we show the detailed derivation of the second Chern number ($C^{(2)}$) of the second Chern crystals in the 4D synthetic translation space. As mentioned in the main text, the second Chern number in the 4D space is defined as

$$\begin{aligned} C^{(2)} &= \frac{1}{32\pi^2}\int \varepsilon_{lmno} B_{lm}(k_x, \Delta x, k_y, \Delta y) B_{no}(k_x, \Delta x, k_y, \Delta y) dk_x d\Delta x dk_y d\Delta y \\ &= C^{(1)}_{k_x,\Delta x} C^{(1)}_{k_y,\Delta y} - C^{(1)}_{k_x,\Delta y} C^{(1)}_{k_y,\Delta x} - C^{(1)}_{k_x,k_y} C^{(1)}_{\Delta x,\Delta y} \end{aligned}, \quad \text{(B1)}$$

where ε_{lmno} is the Levi-Civita symbol, Berry curvature $B_{lm}(k_x, \Delta x, k_y, \Delta y) = \partial_l A_m(k_x, \Delta x, k_y, \Delta y) - \partial_m A_l(k_x, \Delta x, k_y, \Delta y)$ is written in terms of the Berry connection $A_l(k_x, \Delta x, k_y, \Delta y) = \langle u(k_x, \Delta x, k_y, \Delta y) | \partial_l | u(k_x, \Delta x, k_y, \Delta y) \rangle$. Here, $u(k_x, \Delta x, k_y, \Delta y)$ is the periodic wavefunction and each index l, m, n, o takes values of k_x, Δx, k_y, or Δy. We first discuss the first Chern numbers in six 2D subspaces before getting the second Chern number.

Firstly, one can prove that the first Chern numbers in the (k_x, Δx) and (k_y, Δy) subspaces, i.e., $C^{(1)}_{k_x,\Delta x}$ and $C^{(1)}_{k_y,\Delta y}$ are -1. We take $C^{(1)}_{k_x,\Delta x}$ as an example and consider the 2D (k_x, Δx) subspace with a constant k_y and a constant Δy. For conciseness, the subscripts k_y and Δy will be omitted in the following derivations. In the synthetic space, the eigen wavefunctions have fixed relations: the wavefunction at (k_x, Δx, k_y, Δy) can be obtained by translating the one at (k_x, 0, k_y, Δy), i.e. $u_{k_x,\Delta x,k_y,\Delta y}(x, y) = u_{k_x,0,k_y,\Delta y}(x - \Delta x, y)$ which does not depend on the specific form of wavefunction. The Berry connection is then given by

$$\begin{aligned} \mathbf{A}(k_x, \Delta x, k_y, \Delta y) &= i\langle u_{k_x,\Delta x,k_y,\Delta y}(x, y) | \nabla_{k_x,\Delta x} | u_{k_x,\Delta x,k_y,\Delta y}(x, y) \rangle \\ &= i\int_0^a \int_0^a u_{k_x,0,k_y,\Delta y}{}^*(x - \Delta x, y) \nabla_{k_x,\Delta x}[u_{k_x,0,k_y,\Delta y}(x - \Delta x, y)] dxdy \end{aligned}. \quad \text{(B2)}$$

Since $u_{k_x,0,k_y,\Delta y}(x, y)$ is a periodic function in the unit cell, the shift Δx would not affect the integral and $\mathbf{A}(k_x, \Delta x)$ is Δx-independent. A_{k_x} component does not contribute to 2D Berry curvature

$B_{k_x,\Delta x}(k_x,\Delta x)=\nabla_{k_x,\Delta x}\times\mathbf{A}(k_x,\Delta x)$ and subsequently the first Chern number $C^{(1)}_{k_x,\Delta x}$

$$C^{(1)}_{k_x,\Delta x}=\frac{1}{2\pi}\oint_{BZ}\mathbf{A}(k_x,\Delta x)\bullet d(k_x,\Delta x)=\int_a^0 A_{\Delta x}(-\frac{\pi}{a},\Delta x)d\Delta x+\int_0^a A_{\Delta x}(\frac{\pi}{a},\Delta x)d\Delta x. \quad \text{(B3)}$$

Note that the eigen fields for $k_x=\pm\frac{\pi}{a}$ are identical, i.e., $E_{-\frac{\pi}{a},\Delta x}(x,y)=E_{\frac{\pi}{a},\Delta x}(x,y)$. We have $u_{\frac{\pi}{a},\Delta x}(x,y)=u_{-\frac{\pi}{a},\Delta x}(x,y)e^{-i\frac{2\pi}{a}x}$ by using $E_{-\frac{\pi}{a},\Delta x}(x,y)=u_{-\frac{\pi}{a},\Delta x}(x,y)e^{-i\frac{\pi}{a}x}$ and $E_{\frac{\pi}{a},\Delta x}(x,y)=u_{\frac{\pi}{a},\Delta x}(x,y)e^{i\frac{\pi}{a}x}$.

Then,

$$\begin{aligned}A_{\Delta x}(\frac{\pi}{a},\Delta x)&=i\int_{-a/2}^{a/2}u_{-\frac{\pi}{a},\Delta x}{}^*(x-\Delta x,y)e^{i\frac{2\pi}{a}(x-\Delta x)}\frac{\partial}{\partial\Delta x}[u_{-\frac{\pi}{a},\Delta x}(x-\Delta x,y)e^{-i\frac{2\pi}{a}(x-\Delta x)}]dxdy\\&=i\int_{-a/2}^{a/2}\int_{-a/2}^{a/2}u_{-\frac{\pi}{a},\Delta x}{}^*(x-\Delta x,y)\frac{\partial}{\partial\Delta x}[u_{-\frac{\pi}{a},\Delta x}(x-\Delta x,y)]dxdy+i\int_{-a/2}^{a/2}\int_{-a/2}^{a/2}i\frac{2\pi}{a}\left|u_{-\frac{\pi}{a},\Delta x}(x-\Delta x,y)\right|^2dxdy \quad \text{(B4)}\\&=A_{\Delta x}(-\frac{\pi}{a},\Delta x)-\frac{2\pi}{a}.\end{aligned}$$

Plugging into Eq. (B3), we arrive at $C^{(1)}_{k_x,\Delta x}\equiv-1$.

In a similar way, one can prove that the first Chern number in the 2D (k_y, Δy) subspace is -1, i.e.,

$$C^{(1)}_{k_y,\Delta y}(k_x,\Delta x)=\frac{1}{2\pi}\iint_{k_y,\ \Delta y}B_{k_y,\Delta y}(k_x,\Delta x,k_y,\Delta y)dk_y d\Delta y\equiv-1. \quad \text{(B5)}$$

Secondly, one can prove that $C^{(1)}_{k_x,\Delta y}$, $C^{(1)}_{k_y,\Delta x}$ and $C^{(1)}_{\Delta x,\Delta y}$ are 0, which do not depend on the specific form of the 2D wavefunctions. We take the $C^{(1)}_{k_x,\Delta y}$ as an example and consider the 2D (k_x, Δy) subspace with a constant k_y and a constant Δx. The 4D Berry connection is given by

$$\begin{aligned}\mathbf{A}(k_x,\Delta x,k_y,\Delta y)&=i\left\langle u(k_x,\Delta x,k_y,\Delta y)\right|\nabla_{k_x,\Delta x,k_y,\Delta y}\left|u(k_x,\Delta x,k_y,\Delta y)\right\rangle\\&=i\int_0^a\int_0^a u_0{}^*(k_x,x-\Delta x,k_y,y-\Delta y)\nabla_{k_x,\Delta x,k_y,\Delta y}[u_0(k_x,x-\Delta x,k_y,y-\Delta y)]dxdy.\end{aligned} \quad \text{(B6)}$$

Since $u_0(k_x,x,k_y,y)$ is a periodic function in the unit cell, the translation (Δx, Δy) would not affect the integral and $\mathbf{A}(k_x,\Delta x,k_y,\Delta y)$ is Δx- independent and Δy-independent. A_{k_x} component does not contribute to Berry curvature $\mathbf{B}(k_x,\Delta y)=\nabla_{k_x,\Delta y}\times\mathbf{A}(k_x,\Delta y)$ and subsequently the subspace Chern number:

$$C^{(1)}_{k_x,\Delta y}(k_y,\,\Delta x)=\frac{1}{2\pi}\oint \mathbf{A}(k_x,\Delta y)\bullet d(k_x,\Delta y)=\int_a^0 A_{\Delta y}(-\frac{\pi}{a},\Delta y)d\Delta y+\int_0^a A_{\Delta y}(\frac{\pi}{a},\Delta y)d\Delta y\,. \quad \text{(B7)}$$

Combing the equivalence of electric fields at the momentum boundary ($E_z(-\frac{\pi}{a},x,k_y,y)=E_z(\frac{\pi}{a},x,k_y,y)$) and the Bloch theorem ($E_z(k_x,x,k_y,y)=u(k_x,x,k_y,y)e^{k_x x}$), we have $u_0(\frac{\pi}{a},x,k_y,y)=u_0(-\frac{\pi}{a},x,k_y,y)e^{-i\frac{2\pi}{a}x}$. Then Berry connections at the momentum boundary are correlated:

$$\begin{aligned}
&A_{\Delta y}(\frac{\pi}{a},\Delta x,k_y,\Delta y)\\
&=i\iint_{-a/2}^{a/2}u_0^*(-\frac{\pi}{a},x-\Delta x,k_y,y-\Delta y)e^{i\frac{2\pi}{a}(x-\Delta x)}\frac{\partial}{\partial\Delta y}[u_0(-\frac{\pi}{a},x-\Delta x,k_y,y-\Delta y)e^{-i\frac{2\pi}{a}(x-\Delta x)}]dxdy\\
&=i\iint_{-a/2}^{a/2}u_0^*(-\frac{\pi}{a},x-\Delta x,k_y,y-\Delta y)\frac{\partial}{\partial\Delta y}[u_0(-\frac{\pi}{a},x-\Delta x,k_y,y-\Delta y]dxdy\\
&=A_{\Delta y}(-\frac{\pi}{a},\Delta x,k_y,\Delta y).
\end{aligned} \quad \text{(B8)}$$

Plugging into Eq. (B7), we arrive at $C^{(1)}_{k_x,\Delta y}(k_y,\,\Delta x)\equiv 0$. Likewise, we can also prove that $C^{(1)}_{k_y,\Delta x}$ and $C^{(1)}_{\Delta x,\Delta y}$ are 0.

Lastly, $C^{(1)}_{k_x,k_y}$ is zero under time reversal symmetry.

Finally, the second Chern number of the bulk band in the 4D (k_x, Δx, k_y, Δy) space is

$$C^{(2)}=C^{(1)}_{k_x,\Delta x}C^{(1)}_{k_y,\Delta y}-C^{(1)}_{k_x,\Delta y}C^{(1)}_{k_y,\Delta x}-C^{(1)}_{k_x,k_y}C^{(1)}_{\Delta x,\Delta y}\equiv 1\,. \quad \text{(B9)}$$

Therefore, any 2D crystal with a complete band gap is a second Chern crystal in this unique 4D synthetic translation space.

C: Gapless 3D hypersurface modes

The nontrivial topology of the second Chern crystal would manifest itself as the gapless chiral boundary modes when the 4D bulk crystal is truncated to lower dimensional boundaries, i.e. 3D hypersurface, 2D surface or 1D vortex line. In this section, we discuss the case of 3D hypersurface where the crystal is truncated in the x direction [Fig. S3(a)]. As the hypersurface has no discrete translational symmetry along the x direction, k_x is no longer a good quantum number and the surface dispersion is defined on the (Δx, k_y, Δy) hyperplane. The corresponding hypersurface modes emerge as 1D edge modes in the 2D real space, when Δx and Δy are deemed as external parameters. Due to the subspace Chern number $C^{(1)}_{k_x,\Delta x}(k_y, \Delta y) \equiv -1$ for the bulk crystal, chiral gapless dispersion in the Δx direction is expected for any fixed k_y and Δy. To see this, Figs. S3(b)-S3(c) plot the 3D hypersurface dispersions on two typical cut planes. Figure S3(b) shows the dispersion on the (Δx, k_y) plane with Δy = 0. For each fixed k_y, the eigenfrequency of edge mode monotonically decreases as a function of Δx. Since the translation Δy would not affect the surface structure, the hypersurface dispersions are Δy-independent [Figs. S3(c)]. Meanwhile, due to the subspace Chern number $C^{(1)}_{k_x,k_y}(\Delta x, \Delta y) \equiv 0$ for the bulk crystal, the gapped dispersion in the k_y direction is expected for any fixed (Δx, Δy). This is confirmed by the symmetric dispersion in Fig. S3(d), which are also the consequences of time-reversal symmetry. As a whole, the hypersurface band is a 3D chiral sheet on the (Δx, k_y, Δy) plane.

It is noteworthy that in Fig. S3(d), the hypersurface band on the (k_y, Δy) plane are frequency-isolated. Due to the inherently nontrivial topology of bands within the synthetic (k_y, Δy) space, the first Chern number of this hypersurface band is -1. This is confirmed by calculating its numerical Berry curvatures [Fig. S3(e)] whose integral within the synthetic (k_y, Δy) space gives the first Chern number of -1. This nontrivial topology implies that when this 3D hypersurface is further truncated to lower

dimensional boundaries, gapless boundary modes should exist. For example, a gapless corner mode (colored curve in Fig. S3(f)) exists above the projected hypersurface band (translucent red in Fig. S3(f)). This argument also applies for any cut plane with fixed Δy and therefore the dispersion of corner modes is an arched sheet in the (Δx, Δy) space [colored in Fig. 2B of the main text] connecting two projected hypersurface bands [red and blue in Fig. 2B of the main text].

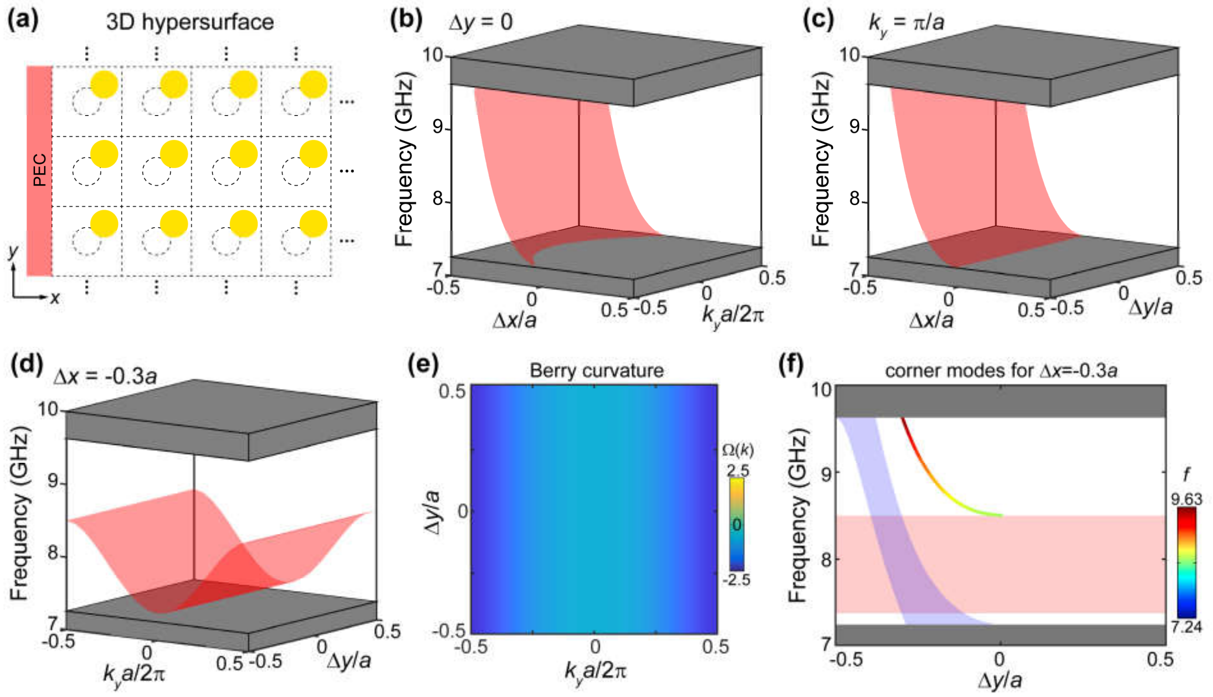

FIG. S3. 3D topological hypersurface modes manifested as 1D edge modes in real space. (a) Schematic of the boundary between the 2D PC with (Δx, Δy) and a PEC on the left. (b-d) Hypersurface mode dispersions on three representative cut planes in the 3D (Δx, k_y, Δy) parameter space, i.e (b) on the (Δx, k_y) cut plane with fixed $\Delta y = 0$, (c) on the (Δx, Δy) cut plane with fixed $k_y = \pi/a$, and (d) on the (k_y, Δy) cut plane with fixed $\Delta x = -0.3a$. (e) Berry curvatures of the hypersurface band in (d) in the (k_y, Δy) space with $\Delta x = -0.3a$, giving first Chern number of -1. (f) Corner mode dispersion for the corner between the 2D PC with ($\Delta x = -0.3a$, Δy) and two PECs on the left and the bottom.

D: Gapless boundary modes at interface between second Chern crystal and trivial insulators

When transverse magnetic modes of 2D PCs are discussed, the PEC is commonly used as an insulator (i.e., band gap material). Therefore, we chose the PEC as a representative example of trivial

insulators in the main text. In this section, we discuss the gapless boundary modes at interface between the second Chern crystal and other trivial insulators. We first discuss the gapless 3D hypersurface modes. Similar to the interface presented in Fig. S3(a), we consider interfaces along the y direction. Figures S4(c)-S4(f) calculate the surface dispersions between the translated PC and one of the four trivial insulators (PEC, perfect magnetic conductor (PMC), untranslated PC, and air). To simplify the discussion, we keep $k_y = \pi/a$ and $\Delta y = 0$. According to the projected bulk bands onto the k_y direction, air behaves as an insulator (due to total internal reflection) since the considered band gap lies below the light line [Fig. S3(b)]. As these four insulators (PEC, PMC, untranslated PC and air) are topologically trivial with $C^{(1)}_{k_x,\Delta x} = 0$, gapless hypersurface dispersion will be expected at the interface. This is confirmed by our numerical results in Figs. S4(c)-S4(f).

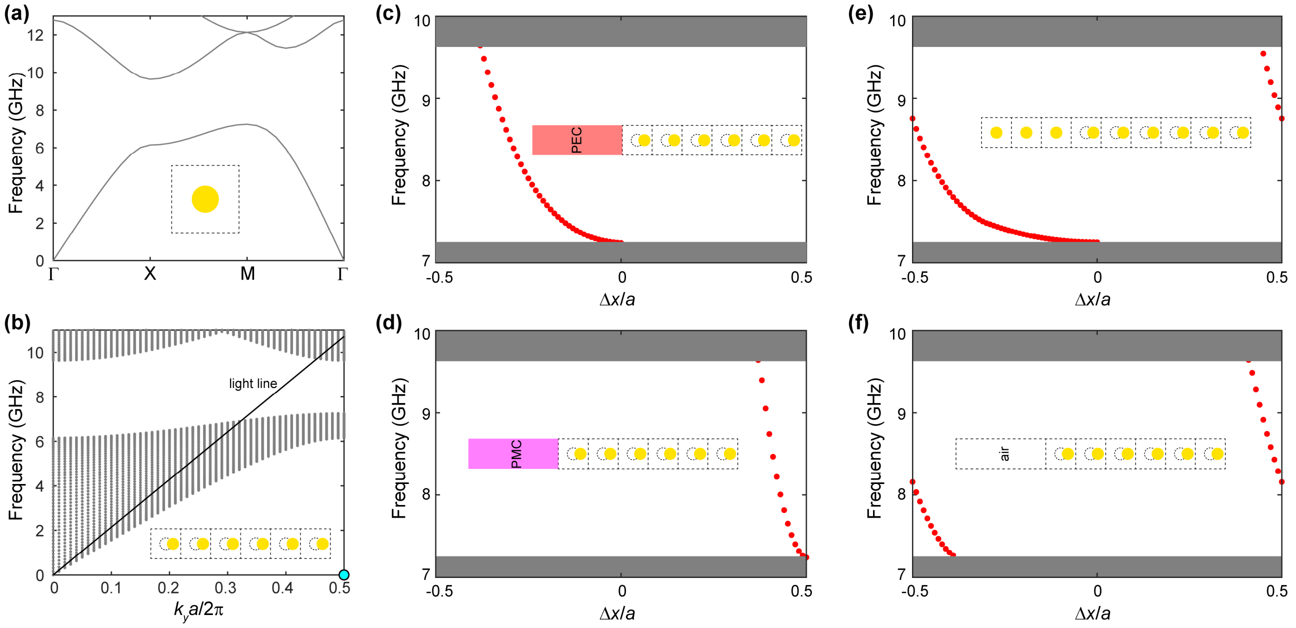

FIG. S4. Gapless hypersurface modes when the second Chern PC is bounded by different trivial insulators. (a) Bulk bands of the second Chern crystals along the high symmetry k-lines of the 2D (k_x, k_y) subspace. (b) Projected bulk bands along the k_y dimension. (c)-(f) Gapless hypersurface modes at the interface between the 2D translated PC on the right and (c) PEC, (d) PMC, (e) the untranslated PC and (f) the air on the left by keeping $k_y = \pi/a$ and $\Delta y = 0$.

Besides of the gapless hypersurface modes, gapless corner modes are also preserved when the second Chern crystal is bounded by other trivial insulators. Instead of the PEC, we form the corner bounded by an untranslated PC [Fig. S5(a)]. As an example, we fix $\Delta x = 0.5a$ in the 2D (Δx, Δy) translation space, and plot the band dispersion along the Δy dimension [Fig. S5(b)]. Gapless corner modes (colored curve) exist outside the projected hypersurface bands (red and blue). The field pattern $|E_z|$ of one representative corner mode at (Δx, Δy) = ($0.5a$, $0.5a$) is plotted in the right panel of Fig. S5(c), showing the strong field confinement around the corner. The existence of corner modes can be also understood by the higher-order topological physics. Under the 2D Zak phase terminology, the untranslated PC (i.e. the PC with (Δx, Δy) = (0, 0)) is characterized by the Zak phase of (0, 0) and the PC with (Δx, Δy) = ($0.5a$, $0.5a$) is characterized by the Zak phase of (π, π). Therefore, the corner mode presented in Fig. S5(c) can be also predicted according to the higher-order topological physics. Therefore, the corner modes presented in Fig. 2 are also related to the corner modes of second-order topological insulators.

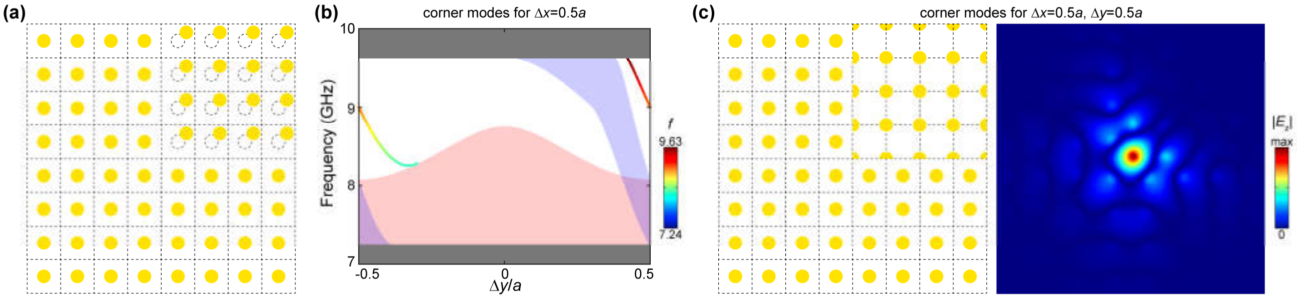

FIG. S5. Gapless corner modes when the translated PC is bounded by an untranslated PC. (a) Schematic of corner consists of the translated PC with (Δx, Δy) bounded by an untranslated PC. (b) 1D gapless dispersion of corner modes of the second Chern crystal as a function of Δy by keeping Δx = $0.5a$. The projected hypersurface bands of the left and bottom boundaries are shaded in red and blue, respectively. (c) Schematic of corner consists of the translated PC with (Δx, Δy) = ($0.5a$, $0.5a$) bounded by an untranslated PC and field pattern $|E_z|$ of the corresponding corner mode.

E: Gapless dislocation modes under different Burgers vectors

As shown in Fig. 3F, one gapless dislocation mode whose frequency increases as Δx exists within the band gap when the dislocation is introduced by a Burgers vector of **B** = (0, a). The number and the monotonous frequency dependence of gapless dislocation modes can be controlled by the Burgers vector of dislocation. To see this, we consider two different dislocation samples which are characterized by Burgers vectors of **B** = (0, -a) and **B** = (0, 2a) [Figs. S6(a) and S6(c)]. When the Burgers vector changes from (0, a) to (0, -a), there is still one gapless dislocation modes traversing the band gap, but the frequencies of dislocation modes monotonously decrease as Δx [Fig. S6(b)]. On the other hand, when the Burgers vector changes from (0, a) to (0, 2a), the frequencies of dislocation modes monotonously increase as Δx. But the number of gapless dislocation modes is changed to be two [marked in red and pink in Fig. S6(d)].

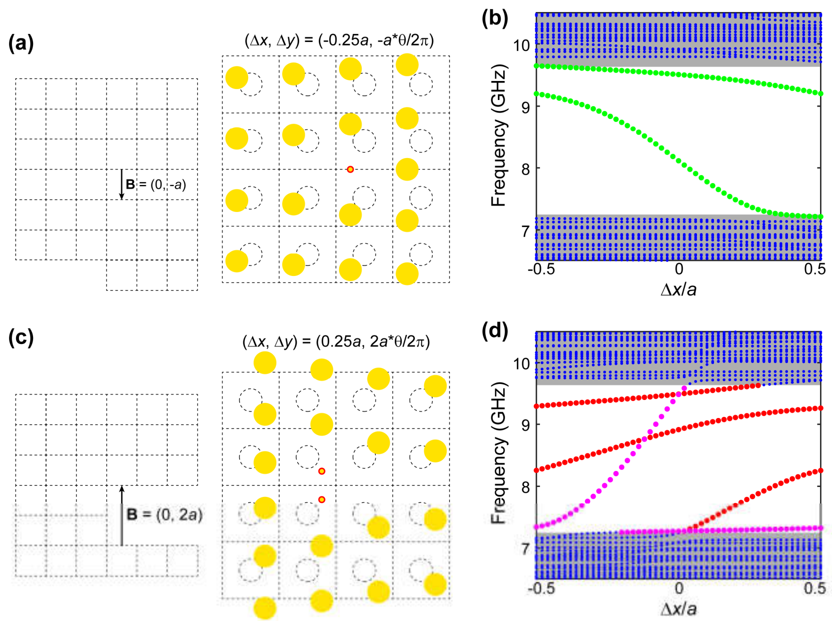

FIG. S6. Gapless dislocation modes for different Burgers vectors. (a & c) Schematic of the dislocation where the translation of each rod is described by (a) (0.25a, -a*θ/2π) and (c) (0.25a, 2a*θ/2π). (b & d) Simulated gapless dislocation modes of the 1D vortex line where the dislocations are characterized by the Burgers vector of (b) **B** = (0, -a) and (d) **B** = (0, 2a).

F: Existence of gapless dislocation modes in spite of the strength of defect

There are always topologically protected dislocation modes in spite of the strength of defect. As a numerical example, we first consider the case of an introduced dielectric rod [Fig. S7]. When the diameters of the defected rod are 2 mm, 5.6 mm and 8 mm, the dispersions of dislocation modes are altered but there are always one gapless dislocation mode dispersion crossing the band gap. As another example, we consider the case of an introduced PEC defect [Fig. S8]. Three PEC defects have the shape of parallelogram with one side length keeping as 9 mm and another side length changing (9 mm, 18 mm and 36 mm). Once again, the mode dispersion of dislocation modes is changed but its gapless feature is preserved.

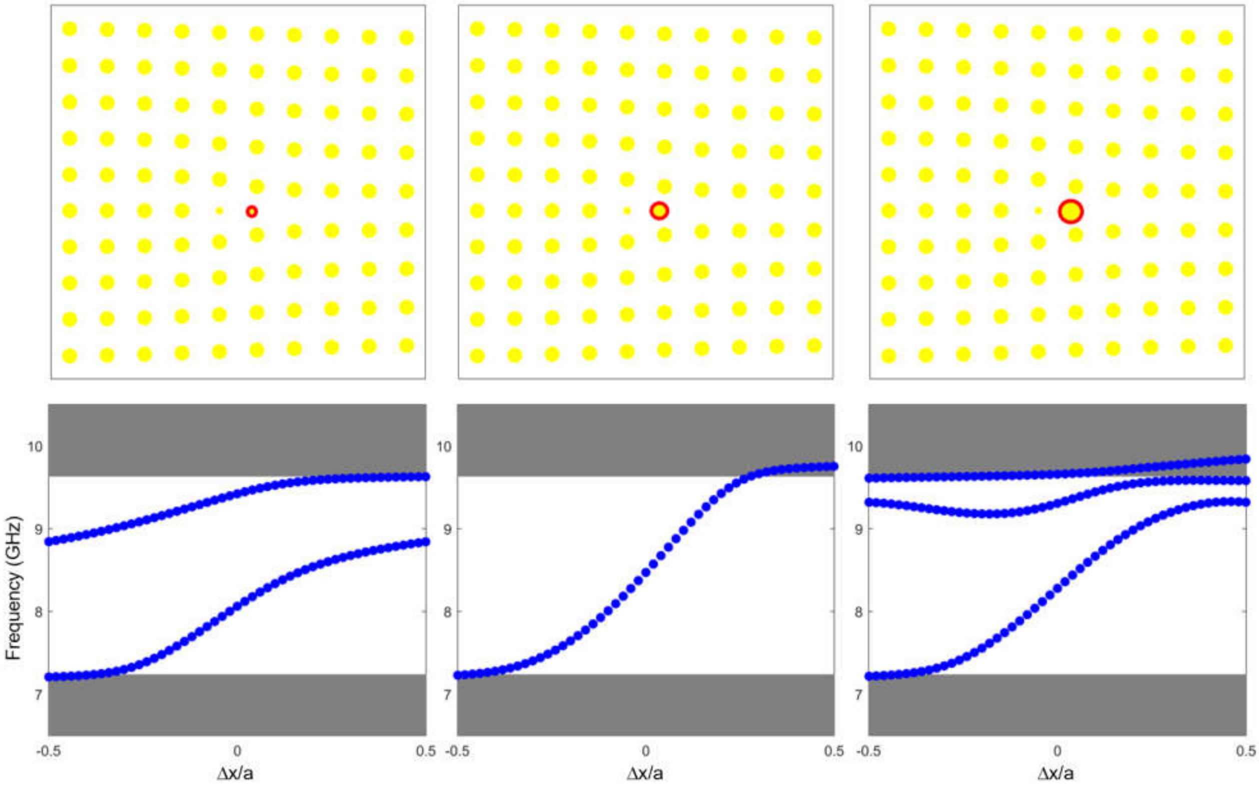

Fig. S7 Simulated frequency spectra for the dislocation samples with an additional dielectric rod. Schematics of the dislocation sample with $\Delta x = 0.5a$ are shown in the upper row. The introduced dielectric rod has a diameter of (left) 2mm, (middle) 5.6mm, and (right) 8mm.

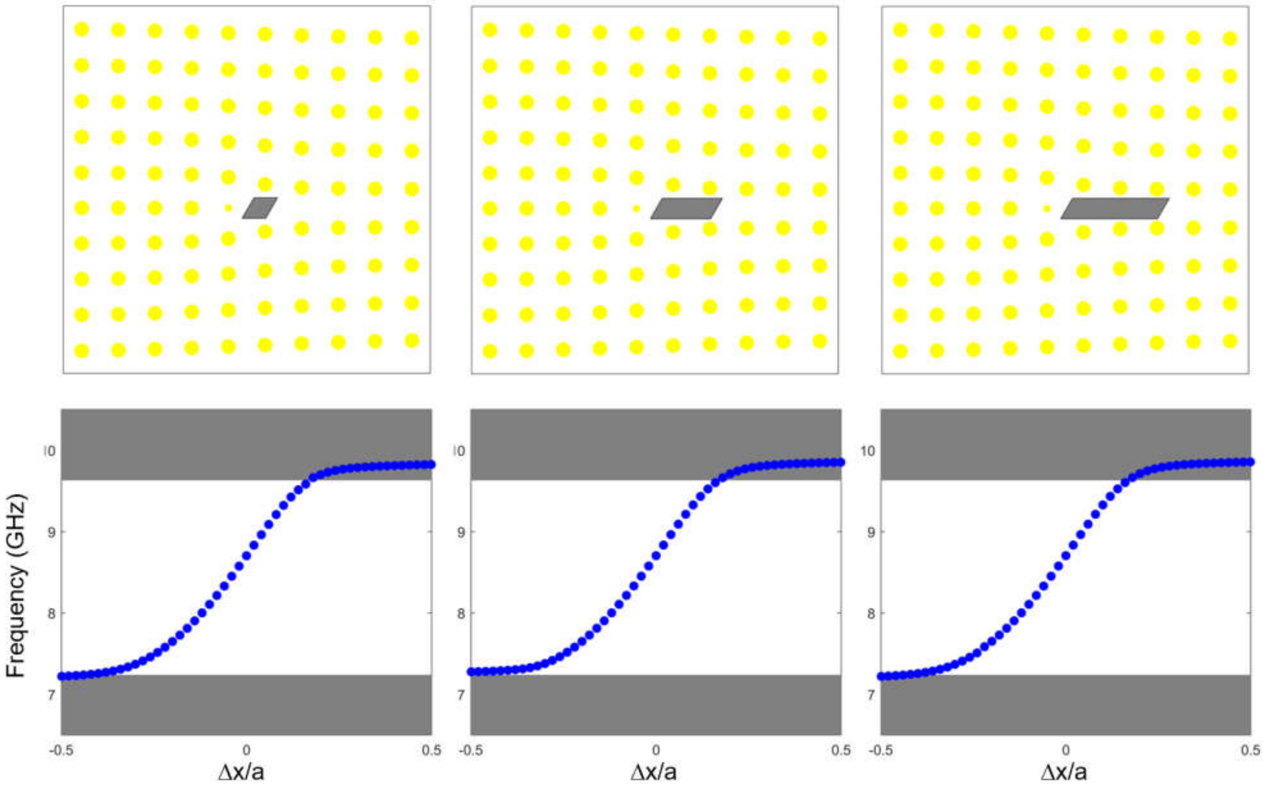

Fig. S8 Simulated frequency spectra for the dislocation samples with a PEC defect. Schematics of the dislocation sample with $\Delta x = 0.5a$ are shown in the upper row. One side length of the PEC is changed (left) 9 mm, (middle) 18 mm, and (right) 36 mm.

G: Gapless corner modes and dislocation modes for the crystals with other lattice types

The topological nature of second Chern crystals in 4D synthetic translation space stems from the nontrivial topology of bulk band within the unique translation space (k_x, Δx, k_y, Δy), rather than the specific form of the wavefunctions or the specific parameters of the crystals. Hence gapless corner modes and gapless dislocation modes are universal for crystals with any lattice, unit cell geometry or material parameter, as long as a complete band gap exists. To see this, we consider two examples.

The first example is an oblique lattice of dielectric rods (with $\varepsilon = 15$) embedded in air [Fig. S9(a)]. Two lattice vectors are $\mathbf{a}_1 = a\mathbf{x}_0$ and $\mathbf{a}_2 = \cos 75^\circ \cdot a\mathbf{x}_0 + \sin 75^\circ \cdot 1.5a\mathbf{y}_0$ where $a = 14$ mm, $\mathbf{x}_0$ ($\mathbf{y}_0$) is the unit vector along the x (y) direction. This oblique lattice PC has the lowest symmetry with $p1$

symmetry and its transverse magnetic modes have a complete band gap from 5.05 to 6.84 GHz. Due to the inherently nontrivial topology, this crystal is a second Chern crystal in the 4D (k_x, Δx, k_y, Δy) space. As a result, gapless corner modes can be found in this band gap when all rods are translated by $\Delta x \cdot \mathbf{a}_1 + \Delta y \cdot \mathbf{a}_2$. This is numerically proved in Fig. S9(b). The 3D hypersurface modes for the left and the bottom PEC boundaries projected onto the (Δx, Δy) plane are shaded in red and blue, respectively. Outside these two projected hypersurface bands are a set of gapless surface modes localized at the corner. To be concrete, we take the cut plane at Δy = -0.3 for example [Fig. S9(c)]. A gapless corner mode (colored curve) exists above the projected hypersurface band (translucent blue). This argument also applies for any cut plane with fixed Δx and therefore the dispersion of corner modes is an arched sheet [colored in Fig. S9(b)] connecting two hypersurface bands. The localized field $|E_z|$ of one representative corner mode at (Δx, Δy) = (-0.2, -0.3) shows strong field confinement around the corner [Fig. S9(d)].

Another manifestation of the nontrivial topology of a second Chern crystal is the gapless dislocation modes along a 1D vortex line. To see this, we consider the sample where the translation of each rod away from its lattice point is $\Delta x \cdot \mathbf{a}_1 + \Delta y \cdot \mathbf{a}_2$ where Δx is fixed at a value between -0.5 and 0.5 while $\Delta y = \theta a/2\pi$. It leads to a dislocation structure near the origin with a Burgers vector of $\mathbf{B} = \mathbf{a}_2$. As indicated by the numerical eigenfrequency spectra as a function of Δx, gapless dislocation modes appear across the whole band gap [Fig. S10(a)]. And the simulated field pattern $|E_z|$ of the representative dislocation mode is given in Fig. S10(b), showing strong field confinement.

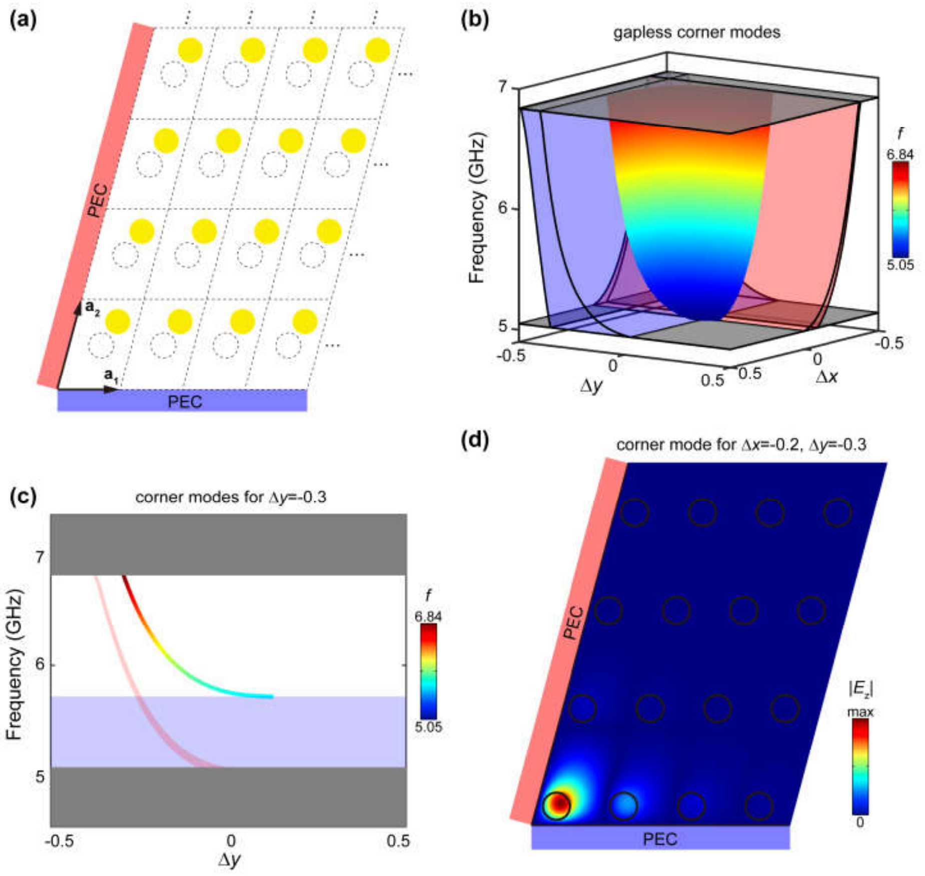

FIG. S9. Gapless corner modes of the second Chern crystal with the oblique lattice. (a) Schematic of the corner between the PC (i.e., the oblique lattice of dielectric rods in the air) and two PEC boundaries. Here, all rods are translated away from the center of the unit cell by $\Delta x \cdot \mathbf{a}_1 + \Delta y \cdot \mathbf{a}_2$. (b) 2D dispersion of the gapless corner modes (shaded in colors) as a function of (Δx, Δy). (c) Corner mode dispersion on the cut plane at Δy = -0.3 in (b). (d) Field pattern $|E_z|$ of the corner mode for the crystal with (Δx, Δy) = (-0.2, -0.3).

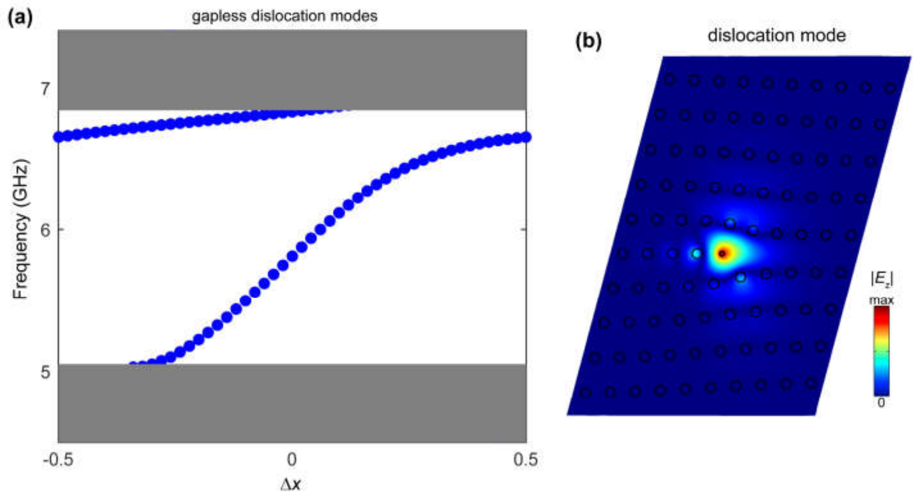

FIG. S10. Gapless dislocation modes. (a) Simulated eigenfrequency spectra in which gapless

dislocation bands are observed. (b) Simulated field pattern $|E_z|$ of the representative dislocation mode.

The other example is a triangular lattice of air holes in dielectric background [Fig. S11(a)]. The radius of air holes is $r = 0.3a$ and the dielectric background is silicon with permittivity of $\varepsilon = 12$. When the transverse electric modes are considered, there is a band gap whose frequency ranges from $0.207c/a$ to $0.274c/a$. By combining two translation dimensions of Δx and Δy along two primitive vectors of $\mathbf{a}_1 = (1,0)$ and $\mathbf{a}_2 = (1/2, \sqrt{3}/2)$, a complete band gap exists in the 4D synthetic translation space and the first bulk band is characterized by the second Chern number of $C^{(2)} = +1$. As a result, gapless corner modes and gapless dislocation modes are expected within the first band gap. Similar to the structures presented in Fig. 3A and Fig. S9(a), we first considered the corner between the PC and two perfect magnetic conductors (PMCs). Gapless corner modes are numerically confirmed in Figs. S11(b) and S11(c). The field pattern $|H_z|$ of the representative corner modes shows the field confinement around the corner [Fig. S11(d)].

To see the gapless dislocation modes, we consider the dislocation with a Burgers vector of $\mathbf{B} = \mathbf{a}_2$. Figure S12(a) shows the dispersion of gapless dislocation modes with the positive group velocity along the $+\mathbf{b}_1$ direction. As an example, we consider one dislocation sample in which each air rod is shifted by $\theta a/2\pi \cdot \mathbf{a}_2$ to its unit cell center. One representative dislocation state with the frequency of $0.233c/a$ have its energy localized near the dislocation point [Fig. S12(b)].

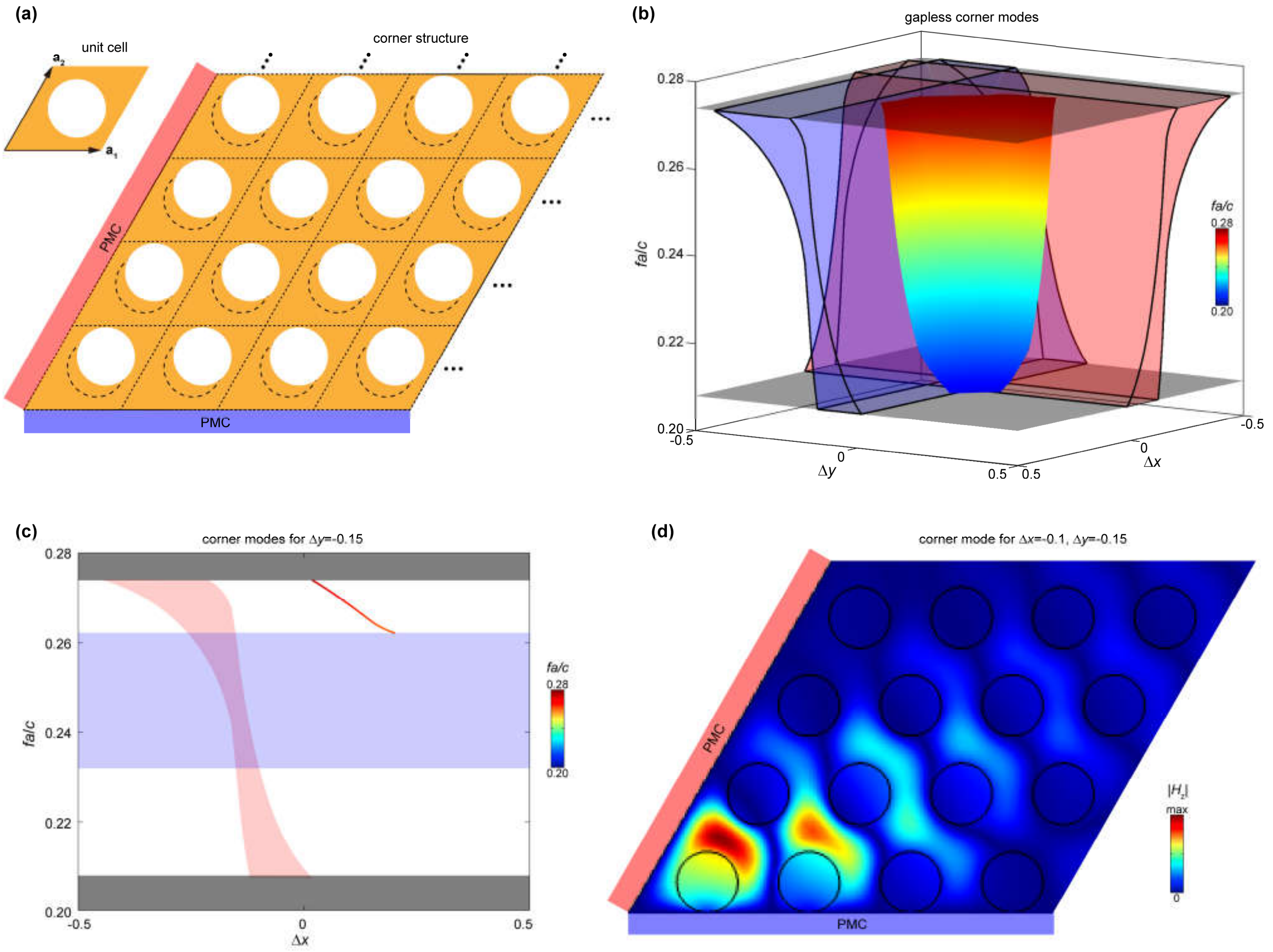

FIG. S11. Gapless corner modes of the second Chern crystal with triangular lattice. (a) Schematic of the corner between the PC and two PMCs. Here, all rods are translated away from the center of the unit cell by ($\Delta x\cdot\mathbf{a}_1$, $\Delta y\cdot\mathbf{a}_2$). (b) 2D dispersion of the gapless corner modes (shaded in colors) as a function of (Δx, Δy). (c) Corner mode dispersion on the cut plane at Δy = -0.15 in (b). (d) Field pattern $|H_z|$ of the corner mode for the crystal with (Δx, Δy) = (0.1, -0.15).

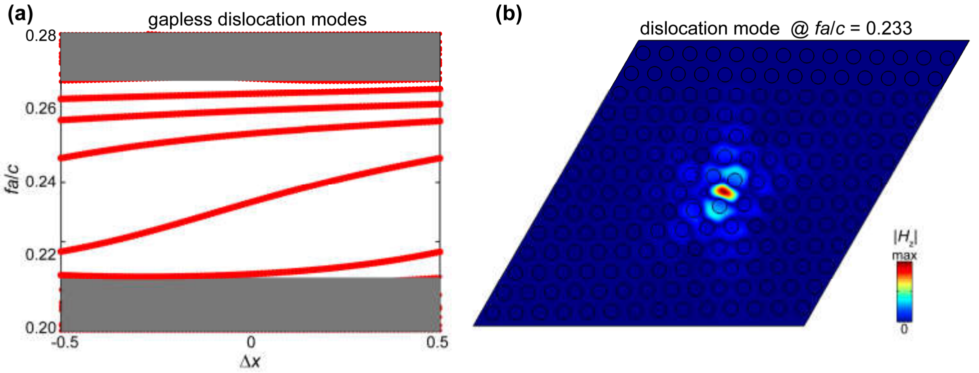

FIG. S12. Gapless dislocation modes. (a) Simulated eigenfrequency spectra in which gapless dislocation bands are observed. (b) Simulated field pattern $|H_z|$ of the representative dislocation mode for the lattice in which each air rod is shifted by ($0\cdot\mathbf{b}_1$, $\theta a/2\pi\cdot\mathbf{b}_2$).

H: Detailed figures for the numerical simulation and experimental measurement

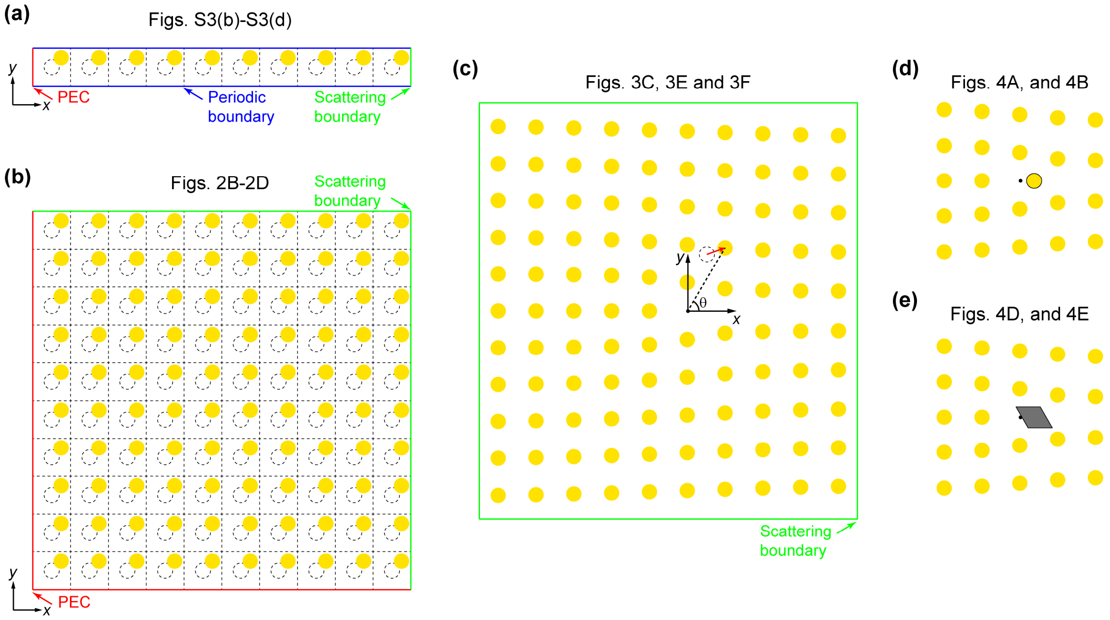

FIG. S13. Models for numerical simulations. The corresponding figures are labelled on the top of each subgraph. In (d) and (e), only part of the structures near the origin are shown to see the introduced defects.

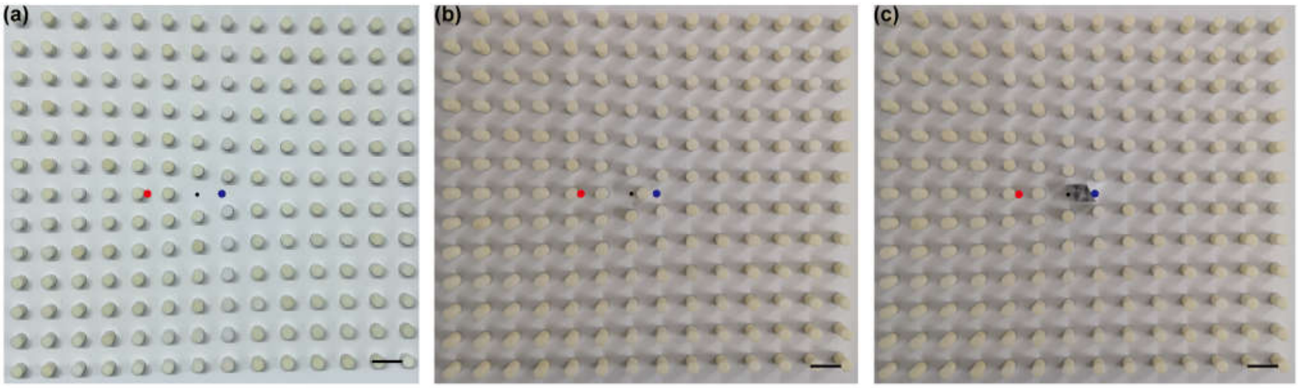

FIG. S14. Samples of the dislocation lattices (a) without and (b, c) with introduced defect. Here, photos of the samples with $\Delta x = 0.5a$ are shown as examples. Around the origin (marked by the black dot), the translations of rods are described by $(0.5a, \theta a/2\pi)$. The centers of the introduced defects (an additional rod and PEC rod) are fixed at (5 mm, 0). The positions of the source and probe antennas are marked by red and blue circles, respectively. Scale bars: 14 mm.

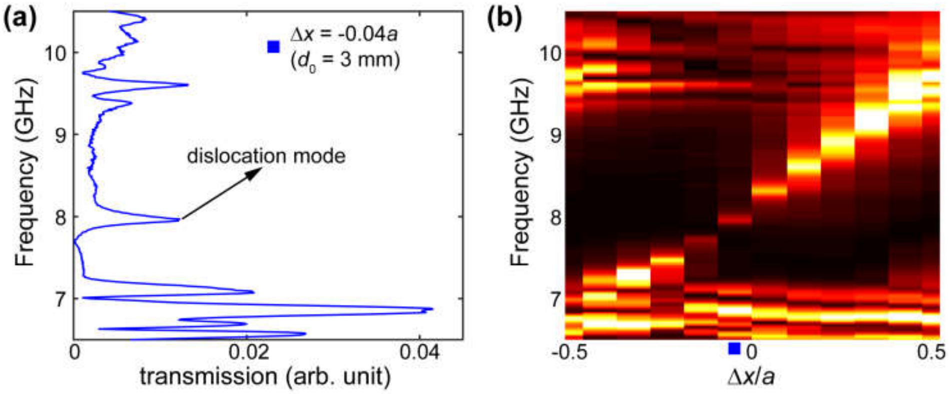

FIG. S15. Measured band dispersion of dislocation modes. (a) The measured transmission spectrum for the dislocation sample with Δx = -0.04a (i.e., the diameter of the first rod to the left of the origin is d_0 = 3 mm). (b) Measured eigenfrequency spectra in which gapless dislocation bands are observed.
